# Case Report: Benralizumab-induced remission in adolescent-onset ANCA-positive eosinophilic granulomatosis with polyangiitis

**DOI:** 10.3389/fimmu.2026.1896474

**Published:** 2026-07-15

**Authors:** Milica Terzic, Aleksandra Plavsic, Stefan Djordjevic, Milos Todorovic, Martina Bosic, Snezana Arandjelovic

**Affiliations:** 1 Clinic for Allergology and Immunology, University Clinical Centre of Serbia, Belgrade, Serbia; 2 Faculty of Medicine, University of Belgrade, Belgrade, Serbia; 3 Division of Pediatric Rheumatology, University Children’s Hospital Tirsova, Belgrade, Serbia; 4 Institute of Pathology, School of Medicine, University of Belgrade, Belgrade, Serbia

**Keywords:** ANCA-associated vasculitis, asthma, benralizumab, biologics, corticosteroid-sparing, eosinophilia, eosinophilic granulomatosis with polyangiitis (EGPA)

## Abstract

Eosinophilic granulomatosis with polyangiitis (EGPA) is a rare antineutrophil cytoplasmic antibody (ANCA)-associated vasculitis characterized by asthma, eosinophilia, and multi-organ involvement. Adolescent-onset disease is uncommon, and evidence guiding biologic therapy in this population remains limited. We report the case of an 18-year-old female with a history of chronic rhinosinusitis and eosinophilic asthma who presented with worsening asthma control, arthralgia, and peripheral eosinophilia (4, 600 cells/μL), with palpable purpura developing during hospitalization. Chest radiography was initially unremarkable, but chest computed tomography revealed peripheral ground-glass opacities and consolidations. Further workup demonstrated eosinophil-rich perivascular infiltrates with karyorrhectic debris without necrotizing vasculitis on skin biopsy, and high-titer anti-myeloperoxidase (anti-MPO) antibodies >200 RU/mL on immunologic testing. The diagnosis of EGPA was established according to the 2022 ACR/EULAR classification criteria with a total score of 10. Initial treatment with high-dose corticosteroids resulted in partial improvement, but the disease relapsed during tapering, indicating corticosteroid dependence. Benralizumab was subsequently introduced, leading to rapid eosinophil depletion, improved pulmonary function, successful corticosteroid reduction, and prompt clinical remission. At 12-month follow-up, the patient remained in complete remission, with normalized pulmonary function, no evidence of new organ involvement, and a marked decline in anti-MPO antibody titers. This case highlights the importance of considering EGPA in adolescents presenting with difficult-to-control eosinophilic asthma and systemic manifestations, and demonstrates that benralizumab can achieve stable, long-term remission with successful corticosteroid reduction even in ANCA-positive disease.

## 1 Introduction

Eosinophilic granulomatosis with polyangiitis (EGPA) is a rare anti-neutrophil cytoplasmic antibody (ANCA)-associated small vessel vasculitis characterized by asthma, peripheral eosinophilia, and variable multi-organ involvement (1, 2). While EGPA predominantly affects adults (3), it is highly uncommon in children and adolescents, leading to poorly defined clinical characteristics and management strategies for this age group (4–6).

Recent advances in understanding eosinophil-driven inflammation have led to the development of biologic therapies targeting the interleukin-5 (IL-5) pathway, with mepolizumab and benralizumab demonstrating efficacy in inducing remission and reducing corticosteroid burden in adult patients with EGPA (7, 8). In cases where the eosinophilic phenotype predominates, characterized by severe eosinophilic asthma, marked peripheral eosinophilia, and tissue eosinophilic infiltration, benralizumab may represents a rational therapeutic choice given its mechanism of direct eosinophil depletion (8). However, clinical experience in pediatric and adolescent-onset EGPA remains limited, as current evidence is largely restricted to isolated case reports, particularly regarding the role of IL-5-targeted therapy in ANCA-positive patients.

We report the case of an 18-year-old female with EGPA, initially presenting as uncontrolled eosinophilic asthma. The development of systemic manifestations prompted a definitive diagnosis, leading to the subsequent achievement of complete remission with benralizumab. The co-occurrence of adolescent onset, ANCA positivity, cutaneous vasculitic features, and an eosinophilic histopathologic pattern without necrotizing vasculitis makes this case particularly instructive. This report aims to: 1) highlight the importance of considering EGPA in adolescents with difficult-to-control eosinophilic asthma who develop systemic manifestations; 2) discuss the diagnostic and phenotypic implications of ANCA-positive disease presenting with a predominantly eosinophilic histologic pattern; and 3) demonstrate that benralizumab can achieve long-term remission with successful corticosteroid reduction in adolescent-onset ANCA-positive EGPA, underscoring that ANCA positivity does not preclude an IL-5-responsive disease phenotype in a population for which clinical evidence remains scarce.

## 2 Case description

### 2.1 Clinical presentation and medical history

An 18-year-old female was admitted to the hospital in June 2024 due to an asthma exacerbation accompanied by atraumatic swelling of the right ankle. One month prior to admission, she had developed arthralgia affecting the small joints of the hands, without joint swelling or morning stiffness.

Her past medical history included surgical correction of scoliosis at age 12 and chronic rhinosinusitis diagnosed at age 14, managed intermittently with hypertonic saline nasal irrigation. Asthma was diagnosed at age 16. At the time of diagnosis, skin prick testing was negative, and the absolute eosinophil count was significantly elevated (2, 000 cells/μL), with no history of nasal polyps or nonsteroidal anti-inflammatory drug sensitivity. There was no relevant family history of autoimmune or atopic disease, and no known environmental or occupational exposures. Sociodemographic and cultural factors were assessed and deemed non-contributory. Treatment with daily inhaled corticosteroids (GINA Step 2) was initiated, with an initially good clinical response, and good adherence to therapy documented throughout this period.

In the months preceding hospitalization, she experienced frequent asthma exacerbations. On admission, the patient reported dyspnea and chest tightness. Vital signs were stable. Physical examination revealed bilateral wheezing on lung auscultation and non-pitting edema in the right perimalleolar region. No hepatosplenomegaly, lymphadenopathy, or neurological deficits were observed. Head, neck, cardiovascular, and skin examinations were otherwise unremarkable.

### 2.2 Initial investigations

Spirometry demonstrated a moderate obstructive ventilatory defect (FVC 73%, FEV_1_ 76%, FEV_1_/FVC ratio 73%, TLC 100%) with a positive bronchodilator response (FEV_1_ increase of 14% post-bronchodilator). Diffusing capacity was preserved (DLCOc 83%, KCOc 96%). The Asthma Control Test (ACT) score was 17, consistent with poor asthma control. Sputum cytology revealed markedly elevated sputum eosinophilia (64%; normal value <3%). Chest radiography showed no abnormalities.

Laboratory evaluation revealed microcytic anemia (hemoglobin 118 g/L, MCV 71.8 fL), leukocytosis (23.2 × 10^9^/L), marked peripheral blood eosinophilia (4, 600 cells/μL), and elevated total IgE (1, 460 IU/mL). Other laboratory parameters, including inflammatory markers, renal and liver function tests, were normal.

Initial management was directed toward treatment of asthma exacerbation. The patient received intravenous methylprednisolone and aminophylline, followed by inhaled corticosteroids and long-acting β_2_-agonist therapy as part of a maintenance and reliever therapy (MART) regimen according to GINA Step 4.

Notably, on the third day of hospitalization, she developed palpable purpuric lesions on the lower legs, a new clinical finding that prompted further diagnostic evaluation.

### 2.3 Diagnostic assessment

Following the appearance of purpuric lesions, systematic organ assessment was undertaken. Abdominal ultrasound showed no abnormalities. Echocardiography revealed mild mitral regurgitation without hemodynamically significant structural or functional abnormalities. Doppler ultrasound of the right ankle confirmed localized subcutaneous edema without evidence of deep vein thrombosis or joint effusion. Urinalysis and proteinuria were normal.

Given the marked eosinophilia, secondary causes and primary hematologic disorders associated with clonal eosinophilia were systematically ruled out. Parasitic infection was excluded based on negative stool and serologic studies. Drug-induced eosinophilia was considered unlikely given the absence of any relevant medication use. Allergic bronchopulmonary aspergillosis was excluded by negative Aspergillus-specific IgE, IgG, and IgM antibodies, as well as negative serum galactomannan. Serum vitamin B12 was within normal limits, and bone marrow aspiration showed an increased proportion of eosinophils with normal granulocytic differentiation and no evidence of an aberrant blast population, consistent with reactive eosinophilia. Genetic testing identified no mutations associated with myeloproliferative neoplasms or other clonal hematologic disorders, excluding a clonal eosinophilic disorder and hypereosinophilic syndrome.

Chest computed tomography (CT) showed peripheral ground-glass opacities and patchy consolidations, particularly in the right upper lobe, findings inconsistent with asthma alone (**Figure 1A**). Histopathological analysis of a skin biopsy performed approximately seven days after the onset of cutaneous lesions demonstrated subepidermal cleft and eosinophil-rich perivascular and interstitial dermal infiltrates with karyorrhectic debris but without evidence of necrotizing vasculitis (**Figures 2A, B**).

**Figure 1:**
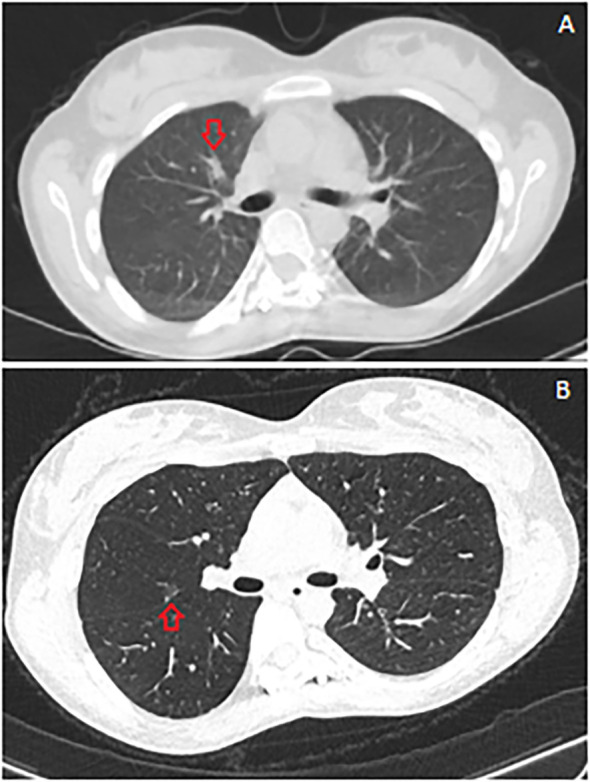
Chest CT imaging. **(A)** Initial chest CT showing irregular area of pulmonary consolidation in the anterior segment of the right upper lobe. **(B)** Follow-up chest CT at 12 months demonstrating significant resolution of previously observed pulmonary infiltrates.

**Figure 2:**
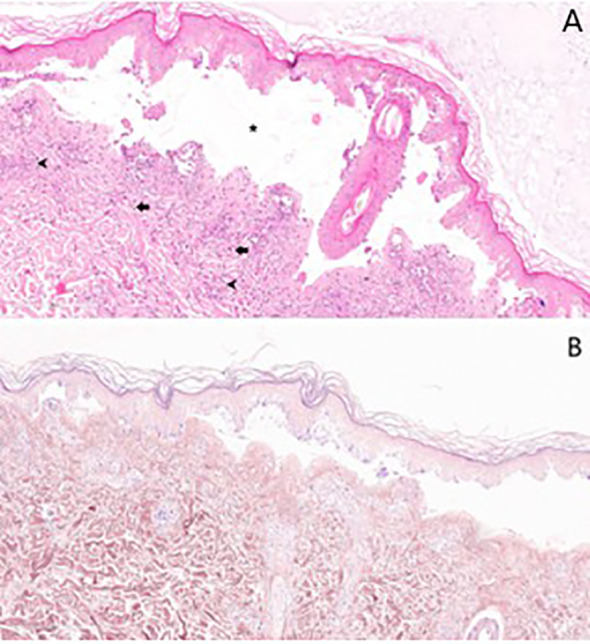
Histopathological examination of a skin biopsy: **(A)** Subepidermal cleft (marked with star) and eosinophil-rich perivascular and interstitial dermal infiltrates (arrows) with karyorrhectic debris (arrowheads) (hematoxylin and eosin staining, magnification 20x); **(B)** No morphological evidence of necrotizing vasculitis (Phosphotungstic Acid-Haematoxylin (PTAH) stain, magnification 20x).

In view of the clinical presentation suggesting systemic vasculitis, immunologic testing was performed, revealing high-titer perinuclear antineutrophil cytoplasmic antibodies (p-ANCA) with markedly elevated anti-myeloperoxidase (anti-MPO) antibodies (>200 RU/mL) and negative anti-proteinase 3 (anti-PR3) antibodies.

In June 2024, the diagnosis of EGPA was established based on the clinical and laboratory findings, including chronic rhinosinusitis, arthralgia, asthma, marked peripheral eosinophilia, positive anti-MPO antibodies, pulmonary involvement, and biopsy-confirmed extravascular eosinophilic infiltrates. These findings fulfilled the 2022 American College of Rheumatology (ACR)/European Alliance of Associations for Rheumatology (EULAR) classification criteria, with a total score of 10, including obstructive airway disease (+3), peripheral eosinophilia ≥1×10^9^/L (+5), and extravascular eosinophil-predominant inflammation on biopsy (+2). The Birmingham Vasculitis Activity Score (BVAS v3) at diagnosis was 7, reflecting worsening wheezing, pulmonary infiltrates, palpable purpura, and new-onset arthralgia. The Five-Factor Score (FFS) was 0, indicating the absence of adverse prognostic factors. The diagnostic workup was associated with several challenges, including the initial normal chest radiography despite active pulmonary pathology subsequently confirmed on chest CT, underscoring the limitations of plain radiography in detecting early parenchymal involvement in EGPA, and the practical delay in obtaining a skin biopsy relative to the onset of purpuric lesions.

### 2.4 Therapeutic intervention

High-dose systemic corticosteroids were initiated, consisting of three intravenous pulses of methylprednisolone (500 mg each), followed by daily intravenous methylprednisolone (60 mg) with gradual tapering and transition to oral prednisone. This regimen led to initial clinical and laboratory improvement, including resolution of purpura and respiratory symptoms, with a decrease in peripheral eosinophil count to 800 cells/μL.

However, approximately seven weeks after diagnosis, attempts to taper prednisone below 30 mg/day were complicated by recurrent asthma exacerbations, arthralgia, and relapse of eosinophilia (3, 800 cells/μL), indicating persistent disease activity and corticosteroid dependence, despite a marked decline in anti-MPO antibody titers from >200 to 4.3 RU/mL. Given the absence of organ- or life-threatening manifestations and persistent eosinophilic disease activity, escalation of therapy with an IL-5 pathway-targeted biologic was considered. As mepolizumab was not available in our country at the time of treatment, and benralizumab was not approved for EGPA but was available for severe eosinophilic asthma, it was introduced off-label as a steroid-sparing agent in August 2024, using the standard dosing regimen for severe asthma (30 mg subcutaneously every four weeks for the first three doses, followed by every eight weeks). A timeline of key clinical events is presented in **Figure 3**.

**Figure 3:**
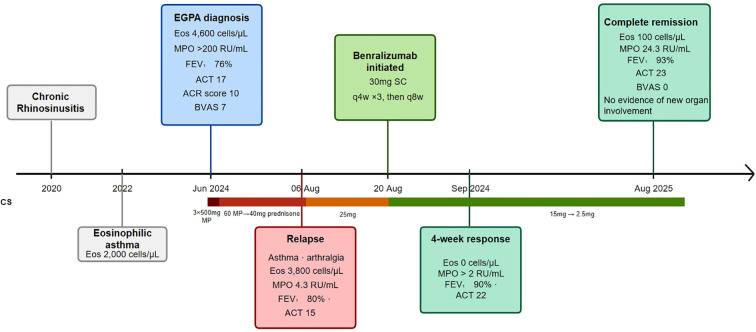
Timeline of the patient’s clinical course. Eos, eosinophils; MPO, anti-myeloperoxidase antibodies; FEV_1_, forced expiratory volume in 1 second; ACT, Asthma Control Test; BVAS, Birmingham Vasculitis Activity Score; SC, subcutaneous; MP, methylprednisolone; q4w, every four weeks; q8w, every eight weeks.

### 2.5 Follow-up and outcomes

Within four weeks of initiating benralizumab, peripheral eosinophil counts decreased to 0 cells/μL, total IgE declined to 156 IU/mL, and anti-MPO antibody titers decreased further to 2 RU/mL. The patient reported marked clinical improvement with resolution of respiratory symptoms and arthralgia. Pulmonary function tests showed improvement, with FEV_1_ increasing to 90% of predicted, and the ACT score improved to 22. Corticosteroid tapering was initiated alongside benralizumab, with prednisone successfully reduced to 15 mg/day within four weeks.

At 12-month follow-up, the patient remained in complete remission on benralizumab, with a peripheral eosinophil count of 100 cells/μL, FEV_1_ of 93% of predicted, ACT score of 23, and BVAS of 0, with no evidence of new organ involvement. Anti-MPO antibody titers were 24.3 RU/mL. A repeat chest CT scan demonstrated significant resolution of previously observed pulmonary infiltrates (**Figure 1B**). Follow-up sputum cytology showed complete resolution of eosinophilic airway inflammation, with no eosinophilia detected. Throughout the follow-up period, prednisone was gradually tapered to 2.5 mg daily without relapse. Benralizumab was well-tolerated throughout, with no adverse drug reactions, tolerability issues, or compliance challenges reported by the patient. The key clinical, laboratory, and treatment parameters across all time points are summarized in **Table 1**.

**Table 1:** Key clinical, laboratory and treatment parameters at diagnosis, at relapse, and during follow-up.

Parameter	At diagnosis June 2024	At relapse August 2024	4 weeks after first dose of benralizumab September 2024	12-month follow-up August 2025
Eosinophils (cells/μL)	4, 600	3, 800	0	100
Total IgE (IU/mL)	1, 460	1, 090	156	344
Anti-MPO antibodies (RU/mL)	>200	4.3	2	24.3
FEV_1_ (% predicted)	76	80	90	93
ACT score	17	15	22	23
BVAS v3	7	3	0	0
Sputum eosinophilia (%)	64	—	—	0
Chest CT	Consolidations and GGO, right upper lobe	—	—	Significant resolution of infiltrates
Treatment	3×500mg MP pulses → iv MP 60mg/day → 40mg/day prednisone	25mg/day prednisone	15mg/day prednisone	2.5mg/day prednisone

Empty cells indicate parameters not measured at that time point. GGO, ground-glass opacities; ACT, Asthma Control Test; BVAS, Birmingham Vasculitis Activity Score; anti-MPO, anti-myeloperoxidase; FEV_1_, forced expiratory volume in 1 second; MP, methylprednisolone.

## 3 Discussion

Although our patient was 18 years of age at the time of diagnosis (the upper limit of adolescence as defined by the World Health Organization), the onset of disease manifestations, including chronic rhinosinusitis at age 14 and eosinophilic asthma at age 16, places this case within the spectrum of adolescent-onset EGPA. To our knowledge, this is the first reported case of adolescent-onset MPO-ANCA-positive EGPA achieving sustained remission with benralizumab as first-line biologic therapy. Given the scarcity of data in this age group, we draw comparisons with both pediatric and adult cohorts throughout this discussion.

EGPA is exceptionally rare, with an estimated incidence of 0.5-4.2 cases per million annually and a prevalence of 10–14 cases per million inhabitants globally (9–11). In children and adolescents, it represents one of the least common forms of pediatric vasculitis, with the three largest reported pediatric series collectively identifying only 36 cases (4–6), with additional isolated case reports in the literature, and the characteristics and optimal management of EGPA in this age group remain poorly defined. While EGPA predominantly affects adults with a mean age at disease onset of approximately 50 years (3, 12), the median age at diagnosis in pediatric series ranged from 12 to 15 years (4–6), highlighting that our patient’s presentation at 18 years represents the older end of the pediatric-adolescent spectrum.

The clinical profile of pediatric EGPA differs from adult-onset disease in several important respects. In adults, asthma typically precedes systemic manifestations by nearly a decade (12, 13), while pediatric patients tend to develop systemic involvement earlier, often during the eosinophilic phase, with a shorter interval between asthma onset and diagnosis (5). In our patient, asthma had been diagnosed only two years prior to the EGPA diagnosis and showed progressive worsening in the months preceding the onset of systemic manifestations, consistent with the well-described pattern of deteriorating asthma control as an early warning sign of systemic disease (12, 13). This shorter prodromal period aligns with the pediatric pattern described by Fina et al. (5) and underscores the importance of maintaining a high index of suspicion for EGPA in young patients with difficult-to-control eosinophilic asthma, even when the duration of asthma is relatively brief. In adolescents, the combination of systemic symptoms such as arthralgia and skin lesions, together with marked peripheral eosinophilia disproportionate to asthma severity, should raise suspicion for EGPA. In our patient, during the acute presentation, prior to the onset of purpura, the differential diagnosis included severe eosinophilic asthma, hypereosinophilic syndrome, and rheumatoid arthritis. The subsequent appearance of palpable purpura, in conjunction with marked peripheral eosinophilia and pulmonary infiltrates, shifted the diagnostic consideration toward EGPA and prompted further immunologic workup, ultimately revealing high-titer anti-MPO antibodies and confirming the diagnosis.

ANCA antibodies are detectable in approximately 30–40% of adult EGPA patients, most commonly as perinuclear ANCA (p-ANCA) with anti-MPO specificity (3). Importantly, ANCA positivity is infrequent in pediatric cohorts, being absent in all patients tested in the series reported by Gendelman et al. (6) and Eleftheriou et al. (4), and present in only 30.7% in the French cohort (5), making our patient’s high-titer MPO-ANCA positivity a particularly unusual feature in this age group.

EGPA is characterized by two partially overlapping pathogenic mechanisms, eosinophil-driven tissue inflammation and ANCA-mediated vasculitic injury, which give rise to two clinically distinct phenotypes (12, 14). The eosinophilic phenotype, typically ANCA-negative, is characterized by tissue eosinophilia and organ damage attributable to eosinophil degranulation, predominantly affecting the heart, lungs, and gastrointestinal tract. The vasculitic phenotype, associated with ANCA positivity, is characterized by more prominent small-vessel inflammation, with higher rates of peripheral neuropathy, renal involvement, and cutaneous purpura (12, 14, 15). However, considerable overlap exists between these phenotypes, and organ involvement does not always correlate with serologic status. Compared with adults, pediatric patients show a higher frequency of cardiac, cutaneous, and gastrointestinal involvement, while neurological manifestations are less common (5, 6).

Our patient exemplifies this phenotypic complexity. The presence of high-titer MPO-ANCA antibodies and palpable purpura suggested a vasculitic component, yet skin biopsy demonstrated eosinophil-rich perivascular infiltrates with karyorrhectic debris without necrotizing vasculitis. Several factors may explain this apparent discordance. First, the biopsy was performed approximately seven days after the onset of purpura, and histopathological findings in small-vessel vasculitis are time-dependent, as active necrotizing vasculitis may resolve within days, leaving predominantly eosinophilic infiltrates as the residual pattern (16). Second, vasculitic lesions in EGPA are often segmental, and sampling variability may further contribute to the absence of definitive histologic evidence of vasculitis (17, 18). Third, pediatric EGPA is more frequently diagnosed during the eosinophilic phase, and biopsies in this population tend to show less vasculitis and more eosinophilic infiltration compared with adults, as reported across the major pediatric series (4–6). Although ANCA positivity reflects neutrophil activation and possible vascular inflammation, histologic evidence of vasculitis is not always demonstrable, and discordance between serology and histopathology has been reported, with some adult ANCA-positive patients lacking histologic vasculitis and a substantial proportion of histologically confirmed cases being ANCA-negative (19). An additional possibility is that the biopsy captured an early or evolving vasculitic lesion. In EGPA, eosinophilic tissue infiltration may precede the development of fully established necrotizing vasculitis, and transitional lesions characterised by eosinophil-rich inflammation with leukocytoclasia but without overt vascular necrosis have been described. Therefore, in the context of palpable purpura and high-titer MPO-ANCA positivity, the observed histopathologic pattern may represent an intermediate stage in the evolution of vasculitic injury.This interpretation is consistent with the view that histopathologic findings in EGPA exist along a continuum, and that the biopsy timing relative to lesion evolution significantly influences the pattern observed.

Importantly, the absence of vascular necrosis on biopsy does not exclude the diagnosis, particularly when characteristic clinical features such as asthma, eosinophilia, and pulmonary involvement are present. Taken together, the clinical and serologic profile of our patient suggests an overlap phenotype in which both pathogenic mechanisms were operationally active, with the eosinophilic component predominating at the time of biopsy, underscoring that ANCA status alone does not reliably distinguish between vasculitic and eosinophilic EGPA phenotypes. The transient decline in anti-MPO antibody titers during clinical relapse, in the setting of rising eosinophilia and worsening asthma, suggests that relapse was predominantly driven by the eosinophilic rather than the vasculitic component. This dissociation between serologic and clinical activity provided further rationale for escalation to IL-5-targeted therapy in this overlap phenotype, underscoring that eosinophil-driven inflammation may remain the dominant pathogenic driver even in ANCA-positive disease.

The markedly elevated sputum eosinophil percentage observed in this patient, together with significant peripheral blood eosinophilia, reflected highly active eosinophil-driven inflammation involving both airway and systemic compartments. While peripheral eosinophilia reflects systemic activity, sputum eosinophilia more directly captures airway type 2 inflammation. Although fractional exhaled nitric oxide (FeNO) was not assessed, the degree of sputum eosinophilia strongly suggests pronounced type 2 immune activation. Eosinophils contribute to tissue injury through the release of cytotoxic granule proteins and proinflammatory mediators, potentially promoting both granulomatous inflammation and vascular damage (12). This marked eosinophilic activation may therefore represent a shared pathogenic driver of airway and systemic manifestations, providing a biologically plausible explanation for the favorable response to targeted eosinophil depletion.

Systemic corticosteroids remain the cornerstone of EGPA treatment, but relapse and corticosteroid dependence are common, underscoring the need for steroid-sparing strategies. Therapeutic strategies in pediatric EGPA remain largely extrapolated from adult data, and all three major pediatric series reported significant corticosteroid dependence and frequent therapy-related complications, including growth retardation, osteoporosis, and Cushing syndrome (4–6). This highlights the particular importance of steroid-sparing strategies in adolescent patients, in whom prolonged corticosteroid exposure carries additional developmental risks.

Therapies targeting the IL-5 pathway have emerged as important options for patients with eosinophilic disease activity (7, 8). According to the 2022 EULAR recommendations, biologic therapy targeting the IL-5 pathway may be considered in patients with relapsing or refractory disease without organ- or life-threatening manifestations (20). When considering biologic therapy in EGPA, the potential influence of ANCA status on treatment response remains an important clinical question. Although EGPA manifests in both ANCA-positive and ANCA-negative forms, current evidence does not support different treatment strategies based solely on ANCA status (21). Benralizumab, a monoclonal antibody targeting the IL-5 receptor α-chain (IL-5Rα), induces rapid and near-complete depletion of eosinophils through antibody-dependent cell-mediated cytotoxicity, a mechanism that distinguishes it from mepolizumab, which neutralizes circulating IL-5 without directly depleting eosinophils (8). In adult EGPA, retrospective cohort studies have demonstrated that benralizumab can induce remission and reduce corticosteroid burden, with efficacy observed regardless of ANCA status (22, 23). However, ANCA positivity has been associated with a higher risk of vasculitic flares during treatment, warranting close monitoring in this subgroup (23). Recent observations suggest that profound eosinophil depletion may influence the clinical expression of EGPA. Ohmura et al. have described patients who developed new-onset or relapsing EGPA during benralizumab treatment, in whom eosinophilic manifestations appeared suppressed while vasculitic features emerged or became more apparent (24). Although our patient differs in that benralizumab was introduced after the diagnosis of EGPA had already been established, these observations highlight the complex interplay between eosinophilic and vasculitic disease mechanisms. Together, they support the concept that eosinophilic and ANCA-mediated inflammatory pathways may evolve independently, and that careful clinical monitoring remains essential even during effective eosinophil depletion.

In the presented case, recurrence of eosinophilia and worsening asthma during corticosteroid tapering indicated persistent disease activity and corticosteroid dependence. The absence of organ- or life-threatening manifestations, reflected by a BVAS of 7 and FFS of 0, supported a biologic steroid-sparing approach over escalation to cyclophosphamide. Benralizumab was introduced off-label using the standard dosing regimen approved for severe eosinophilic asthma, given that it was not approved for EGPA in our country at the time of treatment, and mepolizumab was unavailable. Within four weeks, peripheral eosinophil counts decreased to 0 cells/μL, accompanied by marked clinical improvement, improved pulmonary function, and successful corticosteroid tapering. At 12-month follow-up, the patient remained in complete remission with a BVAS of 0, normalized pulmonary function, and no evidence of new organ involvement. The safety profile of benralizumab in younger patients is supported by long-term data in adolescents and children with severe eosinophilic asthma, demonstrating no unexpected adverse events (25, 26). This case demonstrates that benralizumab can achieve stable, long-term remission with successful corticosteroid reduction in adolescent-onset ANCA-positive EGPA, a population for which clinical evidence remains scarce, and supports its consideration as a first-line biologic option in this setting.Although anti-MPO antibody titers remained detectable at 12 months despite complete clinical remission, this finding is consistent with previous observations demonstrating an imperfect correlation between ANCA titers and disease activity in EGPA. Persistent ANCA positivity does not necessarily indicate ongoing active vasculitis, and treatment decisions should be guided primarily by clinical manifestations rather than serological findings alone. In our patient, the absence of clinical, laboratory, and radiological evidence of active disease supported the interpretation that persistent low-level MPO-ANCA positivity reflected residual immunological activity rather than active vasculitis.

### 3.1 Strengths and limitations

The strengths of this report include detailed clinical, laboratory, radiologic, and histopathologic documentation across multiple time points, as well as long-term follow-up demonstrating sustained remission, with the validity of the observed treatment response supported by objective parameters. This case adds to the limited literature on adolescent-onset EGPA and provides real-world evidence for benralizumab effectiveness in ANCA-positive disease. Limitations include the single-case design, which precludes causal inference and limits generalizability, particularly in pediatric and adolescent populations. Additionally, FeNO was not assessed, which limited the characterization of airway type 2 inflammation.

### 3.2 Clinical implications

This case emphasizes the importance of early recognition of EGPA in adolescents with eosinophilic asthma and evolving systemic manifestations. It also suggests that IL-5 receptor-targeted therapy may be an effective corticosteroid-sparing option in adolescent patients with overlapping eosinophilic and vasculitic features, supporting the concept that eosinophil-driven inflammation remains therapeutically relevant even in ANCA-positive disease.

## 4 Future perspective

Future research should focus on optimal timing and duration of biologic therapy, long-term outcomes, and predictors of treatment response in adolescent-onset EGPA.

## 5 Conclusion

EGPA should be considered in adolescents with difficult-to-control eosinophilic asthma who develop systemic manifestations, even when histopathologic evidence of vasculitis is absent. In this case, benralizumab achieved rapid eosinophil depletion, sustained clinical remission, and successful corticosteroid reduction in an adolescent with ANCA-positive disease, a population for whom optimal therapeutic strategies remain undefined. This observation supports the potential role of IL-5 receptor-targeted therapy across EGPA phenotypes and underscores the need for prospective studies in adolescent-onset disease.

## 6 Patient perspective

The patient reported significant improvement in breathing, daily activity, and quality of life following initiation of benralizumab, and expressed satisfaction with the reduction in corticosteroid use.

## Data Availability

The datasets presented in this article are not readily available because Data sharing is not applicable to this article as it is a case report involving a single identifiable patient. Patient confidentiality and privacy restrictions preclude the sharing of individual clinical data. Requests to access the datasets should be directed to MT, milicaterzic81@gmail.com.
